# Evaluating the disability employment gap and Its determinants: findings from a population-based cohort study in spinal cord injury

**DOI:** 10.3389/fresc.2025.1572158

**Published:** 2025-04-25

**Authors:** Mahesh Sarki, Urban Schwegler, George Austin-Cliff, Mayra Galvis Aparicio, Christine Reuse, Martin W. G. Brinkhof

**Affiliations:** ^1^ Swiss Paraplegic Research, Nottwil, Switzerland; ^2^ Faculty of Health Sciences and Medicine, University of Lucerne, Lucerne, Switzerland; ^3^ Institute of Vocational Integration (ParaWork), Swiss Paraplegic Center, Nottwil, Switzerland

**Keywords:** labor market participation, spinal cord injury, disability employment gap, general population, longitudinal

## Abstract

**Introduction:**

The disability employment gap (DEG) is instrumental in monitoring social progress and employment inequalities. This study evaluated the DEG and its determinants among people with spinal cord injury (SCI) in Switzerland.

**Methods:**

Employment data from three consecutive population-based surveys were analyzed and compared with the general Swiss population, matched according to sex, age, year, and region of residence. Mixed-effects Poisson regression modelling was applied to evaluate the determinants of labor market participation (LMP) and derive marginal predictions for the DEG.

**Results:**

DEGs decreased over calendar time, with individuals with complete tetraplegia exhibiting the most substantial reduction (2012: −37%, 2022: −25%); however, their probability of LMP in 2022 remained 25% lower than those with incomplete paraplegia. The DEG marginally increased among those with the fewest years of education (0–9) (2012: −48.1%, 2022: −49.2%). Regional disparities were also observed.

**Conclusions:**

Our findings indicate improved LMP opportunities in the Swiss SCI population, but also highlight the need to promote access to quality employment opportunities, vocational education, and training programs for severely injured individuals with low education. Additionally, efforts should be made to ensure equal LMP prospects across Switzerland.

## Introduction

Employment gaps between individuals with disabilities and the general population may indicate poor socioeconomic inclusion and highlight inequalities within a country (1). At the societal level, persistent disability employment gaps (DEGs) contribute to a loss of productivity, reduced tax revenues, and increased social security expenses (2). At the individual level, they may lead to financial instability, a lower quality of life, poor self-esteem, and poor health outcomes (1, 3). Consequently, the DEG was included as a key indicator in the European Pillar of Social Rights' Social Scoreboard to monitor social progress in the European Union (4). The DEG is crucial for creating political momentum to optimally plan labor market participation (LMP) policies and vocational integration programs aimed at reducing employment disparities and ensuring social equity.

The incidence of LMP is lower in people with spinal cord injury (SCI) than in the general population. Globally, the DEG varies widely between countries, ranging from 15% to 55% (5). Although Switzerland had the highest LMP rate among persons with SCI in 2017, this rate remained approximately 20% lower than that of the general population (5). Switzerland has a federalized system, in which regional units (i.e., cantons) with their disability insurance offices are responsible for the organization and funding of vocational integration service delivery (6). Due to this federalized system, the actual implementation of the national disability insurance law varies across Swiss cantons, leading to regional differences in the accessibility and quality of vocational integration services. Therefore, it is essential to evaluate DEGs segregated by region in Switzerland.

SCI can serve as a valuable benchmark for examining the overall DEG between individuals with disabilities and the general population because it results in a range of impairments that potentially limit an individual's work abilities and ability to follow workplace norms (7). Moreover, environmental barriers, such as poorly accessible workplaces, lack of transportation services, negative social perception of competence, and non-inclusive labor market policies, have a considerable impact on the LMP (3, 8), making SCI a good example for studying the DEG as an indicator of employment disparities in different countries.

Currently, there is a lack of robust evidence concerning the development of the DEG in persons with SCI. Most studies that have examined the DEG are cross-sectional (5, 9), while others that have used cross-sectional or panel data to assess longitudinal changes in LMP have not included comparisons with the general population (10, 11). Furthermore, the individual, social, contextual, and performance-related determinants of LMP in individuals with spinal cord injury are extensively documented (3, 12, 13). However, these parameters are frequently not aligned with those of the general population, thereby limiting the validity of standardized DEG estimates. Valid DEG estimates and their social and clinical determinants, such as education level, region of residence, and SCI severity, are crucial for identifying subgroups that require special attention when establishing policies that promote equity in LMP and reduce social disparities (14, 15). In a recent study using longitudinal panel data of the Swiss Spinal Cord Injury (SwiSCI) cohort study, we provided an initial overview of DEGs among people with SCI in comparison to the general Swiss population (16). The present study aims to comprehensively examine the factors influencing DEGs and generate adjusted DEG estimates for the 2012–2022 period, stratified by SCI severity, educational attainment, and region of residence.

## Methods

### Study design and setting

This study used survey data from the population-based Swiss Spinal Cord Injury Cohort Study (SwiSCI) collected in 2012, 2017, and 2022 (17, 18). The SwiSCI survey evaluates comprehensive data on health and functioning in individuals with traumatic or non-traumatic SCI who reside in Switzerland and are 16 years of age or older at the time of the survey. The SwiSCI study was approved by the regional ethics committees of the four participating SCI-specialized rehabilitation facilities. Written informed consent was obtained from all study participants. The SwiSCI community surveys were conducted in line with the ethical principles outlined in the Declaration of Helsinki (18).

We also used LMP data from the general Swiss population obtained from the Swiss Labor Force Survey (SLFS), stratified by age, sex, region of residence, and SwiSCI survey year (i.e., 2012, 2017, and 2022) (16). The SLFS annually collects data from approximately 100,000 Swiss residents, providing a statistically representative subset of the population (19).

### Study population

The SwiSCI study focuses on individuals with acquired traumatic or non-traumatic SCI. Participants are recruited through registries maintained by specialized SCI rehabilitation centers, the Swiss Paraplegic Association (i.e., the organization for people with SCI in Switzerland), and ParaHelp (i.e., the specialized home care institution for persons with SCI in Switzerland). The SwiSCI study excludes individuals with congenital SCI, neurodegenerative disorders, and Guillain-Barré syndrome. In the current study, we additionally excluded individuals with recovered SCI status or unknown SCI type, while focusing on those who participated in one or more of the three SwiSCI community surveys and were within the Swiss working-age range. The statutory working age in Switzerland is 16–64 for men and 16–63 for women.

### Outcome measures

Participants' responses on the multiple-option question regarding the current working situation were dichotomized into “paid work” (including “working for wages with an employer”, “paid vocational training”, and “self-employed”) vs. “no paid work” (including “housework”, “unemployed”, “unpaid work in family business”, “unpaid vocational training or retraining”, “student”, “receiving a disability or another pension”, “retired due to health condition”, “retired due to age”, “sheltered work^1^”, and “other”). If participants selected options from both paid work (e.g., “self-employed”) as well as unpaid work (e.g., housework), they were assigned to “paid work”. The definition of paid work used in this study is consistent with that used by SLFS (19). The DEG was defined as the difference in the LMP rate between persons with SCI and the general population, standardized for age, sex, survey year, and region of residence, and is indicated as a percentage (%).

### Independent variables

Sociodemographic predictor variables included sex (male vs. female), years of education, age at the time of survey, and region of residence. Years of education was categorized into four groups (i.e., 0–9, 10–12, 13–16, and ≥17 years) corresponding to the International Standard Classification of Education (20). Similarly, age at survey was classified into four groups (i.e., 16–24, 25–39, 40–54, and 55–63/64 years) in alignment with the age group classification used by the SLFS (21). Information on participants’ region of residence was collected based on the postal code of the participants' postal address. Postal codes are linked to the greater geographical regions of Switzerland based on the Nomenclature of Territorial Units for Statistics, providing a standard geographical referencing of Switzerland's administrative divisions for statistical purposes (16). Switzerland is sub-grouped into seven greater regions of residence (22), comprising the Lake Geneva Region (cantons: Vaud, Valais, and Geneva), Ticino (canton: Ticino), Zurich (canton: Zurich), Northwestern Switzerland (cantons: Basel-City, Basel-Country, and Argovia), Eastern Switzerland (cantons: Glarus, Schaffhausen, Appenzell Outer-Rhodes, Appenzell Inner-Rhodes, St. Gallen, Grisons, and Thurgovia), Central Switzerland (cantons: Lucerne, Uri, Schwyz, Obwald, Nidwald, and Zug), and the Midland (cantons: Bern, Freiburg, Solothurn, Neuchâtel, and Jura) (16, 22).

SCI-related predictor variables included the cause (i.e., traumatic vs. non-traumatic) and the severity of injury. SCI severity was determined based on SCI lesion level (paraplegia vs. tetraplegia) and lesion completeness (incomplete vs. complete). For participants who were recruited through the four specialized SCI rehabilitation centers, the primary source of these data was medical records, where lesion completeness is defined based on the American Spinal Injury Association Impairment (ASIA) Scale (23). The limited number of individuals recruited through the Swiss Paraplegic Association or ParaHelp lacked SCI-related clinical data; therefore, their SCI characteristics were collected through self-reports. Brinkhof et al. established that self-reported demographic data and SCI characteristics exhibited a high degree of consistency with the corresponding medical record data (24).

### Data analysis

All analyses were conducted using STATA version 18.0 (StatCorp LLC, College Station, TX, USA). Mixed-effects Poisson regression modelling was employed with the general population-standardized employment rate serving as an offset. Univariate Poisson regression model estimates were used to calculate the crude LMP rate in SCI. Crude rate differences (i.e., DEG with 95% CI) were determined by calculating the difference in LMP rates between people with SCI and the general population. An adjusted Poisson regression model was used to evaluate the determinants of LMP. The goodness of fit of the regression models was evaluated using deviance and Pearson chi-squared test. Nonresponse propensities were estimated using a generalized linear mixed model, with the addition of a personal identifier as a random effect. The nonresponse weights were calculated as the inverse of the probability of participation in the survey, which was derived using multivariable mixed-effect modelling.

To examine the development of DEGs over the past decade, we conducted subgroup analyses and *post hoc* (data-driven) interaction tests for different subgroups using mixed-effects Poisson regression modelling. Interactions between potential predictor variables (SCI severity, education, and region of residence) and calendar time (survey) with respect to the development of DEGs over time were assessed. The interaction tests were controlled for stable (i.e., sex) and temporal control variables (i.e., age, and time since SCI), as well as for the personal identifier (SwiSCI ID) as a random-effect variable to account for within-person change in individuals participating in multiple SwiSCI community surveys. The general population-standardized LMP rate was included as an offset in the regression model. The Stata margins command was used to estimate the adjusted marginal DEG.

## Results

Data from 3,060 observations representing 2,006 working-age individuals were used from three SwiSCI community surveys. Of these working-age individuals, 1,230 participated in one, 495 in two, and 281 in all three of the SwiSCI surveys. Nearly three-fourths (73%) of the participants were male and 85% had a traumatic SCI (Table 1). Incomplete paraplegia was the most common SCI severity level (38%). Overall, LMP rates were 56% in 2012, 61% in 2017, and 65% in 2022.

**Table 1:** Sample characteristics and LMP rates in people with SCI and in relation to the general Swiss population.

Parameters	*N* (%)	LMP rate in SCI population (%)	LMP rate in the general population (%)^a^	Crude disability employment gap (%), (95% CI)
Survey year				
2012	1,194 (39.0)	56.1	82.8	−26.7 (−30.9 to −22.3)
2017	1,028 (34.0)	61.1	83.5	−22.4 (−27.4 to −17.5)
2022	841 (27.5)	64.7	83.1	−18.5 (−24.4 to −13.2)
Sex				
Female	850 (27.8)	52.1	75.7	−23.5 (−28.2 to −18.4)
Male	2,213 (72.3)	63.2	86.0	−22.8 (−26.1 to −19.4)
Age (years)				
16–24	76 (2.5)	36.8	62.6	−25.8 (−37.2 to −9.2)
25–39	634 (20.7)	69.6	88.2	−18.6 (−24.9 to −11.7)
40–54	1,306 (42.6)	64.3	88.5	−24.2 (−28.5 to −19.7)
55–63/64	1,047 (34.2)	50.8	74.8	−23.9 (−28.1 to −19.4)
Education (years)				
0–9	177 (6.0)	31.9	80.7	−48.9 (−56.3 to −39.1)
10–12	592 (20.0)	44.7	81.9	−37.3 (−42.4 to −31.5)
13–16	1,442 (48.6)	63.0	83.3	−20.3 (−24.3 to −16.0)
17+	755 (25.5)	75.9	84.5	−8.6 (−14.6 to −2.1)
SCI severity				
Incomplete paraplegia	1,119 (37.7)	64.7	82.4	−17.7 (−22.2 to −12.7)
Complete paraplegia	944 (31.8)	62.9	83.7	−20.8 (−25.7 to −15.5)
Incomplete tetraplegia	594 (20.0)	54.3	82.9	−28.5 (−34.2 to −22.2)
Complete tetraplegia	313 (10.5)	51.1	84.9	−33.8 (−41.2 to −25.2)
Time since SCI (years)				
≤5	546 (18.4)	54.7	82.3	−27.6 (−33.6 to −21.0)
6–15	969 (32.6)	61.3	84.1	−22.8 (−27.6 to −17.7)
16–25	683 (23.0)	64.1	84.8	−20.6 (−26.4 to 14.3)
26+	775 (26.1)	59.6	81.1	−21.6 (−26.8 to −15.8)
Etiology				
Traumatic	2,567 (84.1)	61.2	83.7	−22.4 (−25.4 to −19.3)
Non-traumatic	486 (15.9)	54.0	80.3	−26.4 (−32.6 to −19.4)
Region of residence				
Lake Geneva region	521 (17.0)	49.9	77.4	−27.5 (−33.2 to −21.0)
Midland Switzerland	800 (26.1)	60.4	85.0	−24.6 (−29.8 to −19.0)
Northwestern Switzerland	409 (13.4)	56.9	82.7	−25.9 (−32.8 to 18.0)
Zurich	348 (11.4)	62.9	84.6	−21.6 (−29.5 to −12.6)
Eastern Switzerland	384 (12.5)	66.3	85.7	−19.4 (−27.2 to −10.7)
Central Switzerland	355 (11.6)	66.3	85.5	−19.1 (−27.1 to −10.0)
Ticino	137 (4.5)	60.4	79.4	−19.1 (−30.8 to −4.6)

CI, confidence interval; LMP, labor market participation; SCI, spinal cord injury; N, total number of participants.

^a^ The reference LMP rate for each group, classified by SCI lesion level, time since SCI and SCI etiology, was determined by calculating the average LMP rate of the general population, standardized for age, sex, region, and survey year specific to that group.

Individuals with complete tetraplegia had a 24% (IRR: 0.76; 95% CI: 0.66–0.89) lower likelihood of LMP than those with incomplete paraplegia. Similarly, fewer years of education was associated with a decreased probability of LMP; for instance, individuals with 0–9 years of education had a 55% lower probability of LMP (IRR: 0.45, 95% CI: 0.34–0.61) than those with 17 or more years of education. The region of residence also predicted LMP (*X*^2^ = 14.07, *p* = 0.03); in particular, people residing in Central and Eastern Switzerland as well as in Ticino had a 25%, 24%, and 24% higher likelihood of LMP, respectively, compared to those from the Lake Geneva region. Individuals aged 25–39 years had a 15% higher likelihood of LMP (IRR: 1.15, 95% CI: 1.05–1.27) compared to those in the oldest age range (55–63/64 years), while sex was not predictive of LMP (Table 2). The regression model presented in Table 2 was used to calculate adjusted marginal predicted LMP rates in the SCI sample, demonstrating that LMP rates increased from 57% in 2012, to 61% in 2017, and 66% in 2022, and that DEG decreased from −26% in 2012, to −22% in 2017, and −17% in 2022.

**Table 2:** Determinants of LMP in people with SCI: results from unadjusted and adjusted mixed-effects poisson regression models.

Parameters	Univariable model		Multivariable model^a^	
	IRR (95% CI)	*p*-value	IRR (95% CI)	*p*-value
Survey year		0.001		0.13
2017	1.04 (0.96–1.11)		1.03 (0.97–1.11)	
2022	1.15 (1.07–1.23)		1.08 (1.00– 1.16)	
2012	1		1	
Sex		0.10		0.19
Female	0.93 (0.85–1.02)		0.94 (0.86–1.03)	
Male	1		1	
Age (years)		0.001		0.03
16–24	0.90 (0.65–1.26)		0.93 (0.66–1.32)	
25–39	1.20 (1.09–1.32)		1.15 (1.04–1.27)	
40–54	1.12 (1.03–1.22)		1.09 (1.01–1.18)	
55–63/64	1		1	
SCI severity		<0.001		<0.001
Complete paraplegia	0.95 (0.88–1.03)		0.93 (0.86–1.01)	
Incomplete tetraplegia	0.83 (0.75–0.93)		0.82 (0.74–0.91)	
Complete tetraplegia	0.76 (0.66–0.89)		0.75 (0.65–0.86)	
Incomplete paraplegia	1		1	
Time since SCI (years)		0.10		0.03
≤5	0.90 (0.81–1.01)		0.87 (0.78–0.97)	
6–15	0.98 (0.89–1.08)		0.90 (0.82–0.99)	
16–25	1.03 (0.94–1.13)		0.97 (0.89–1.06)	
>25	1		1	
Education (years)		<0.001		<0.001
0–9	0.44 (0.34–0.58)		0.45 (0.34–0.61)	
10–12	0.60 (0.54–0.68)		0.64 (0.57–0.72)	
13–16	0.85 (0.79–0.91)		0.88 (0.82–0.94)	
≥17	1		1	
Region of residence		0.10		0.03
Midland region	1.16 (1.02–1.31)		1.20 (1.05–1.36)	
Northwestern Switzerland	1.07 (0.92–1.24)		1.11 (0.96–1.29)	
Zurich	1.17 (1.01–1.36)		1.15 (0.99–1.33)	
Eastern Switzerland	1.20 (1.04–1.38)		1.24 (1.08–1.42)	
Central Switzerland	1.21 (1.05–1.40)		1.25 (1.08–1.43)	
Ticino	1.12 (0.90–1.40)		1.24 (1.02–1.52)	
Lake Geneva Region	1		1	

IRR, incidence rate ratio; CI, confidence interval; SCI, spinal cord injury.

^a^ Survey year, time since SCI, sex, age, SCI severity, years spent in education and region of residence were adjusted.

**Figure 1:**
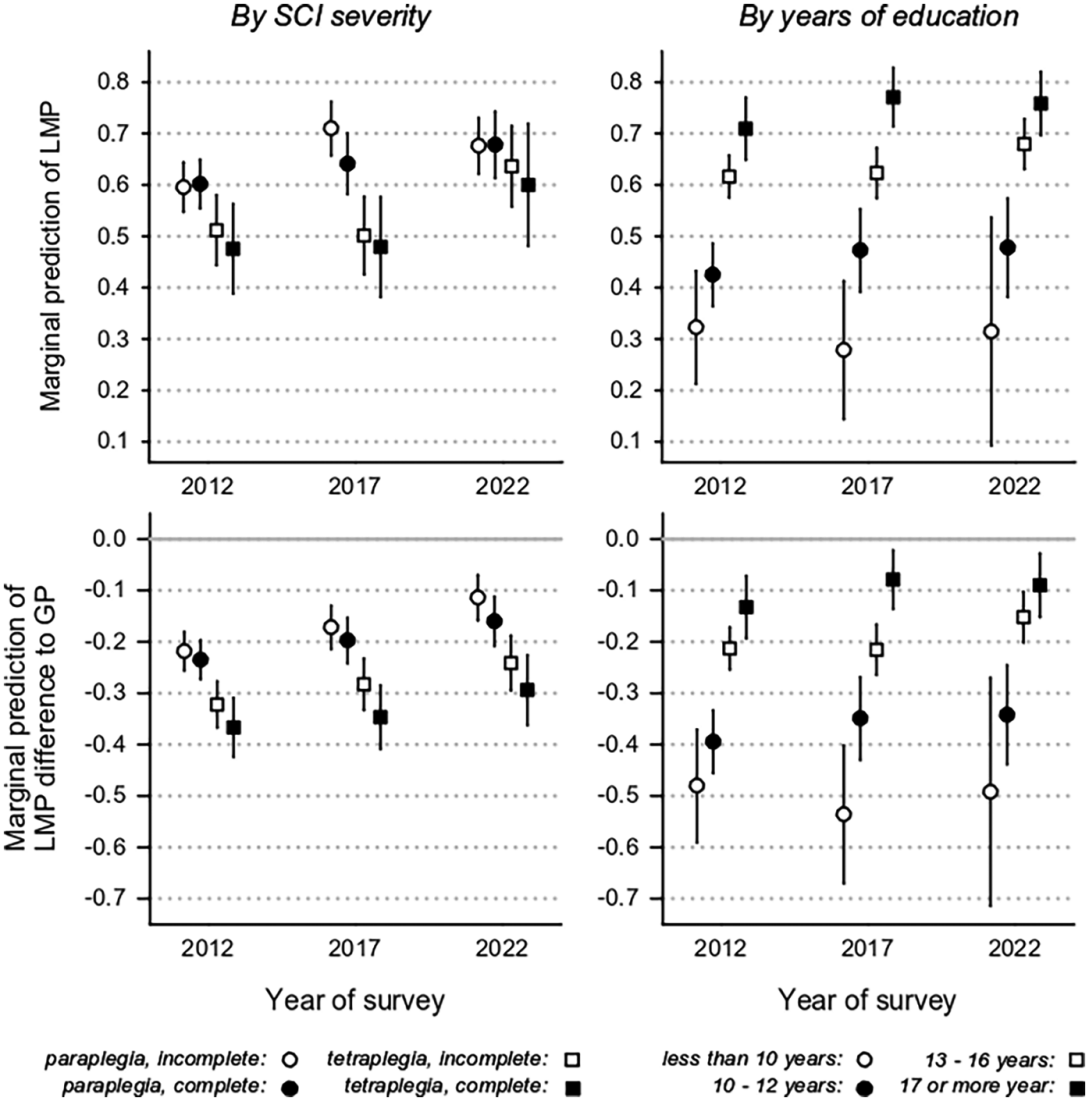
Marginal prediction of labor market participation (LMP) and LMP difference with respect to the general population (disability employment gap) segregated by level of SCI severity and education.

**Figure 2:**
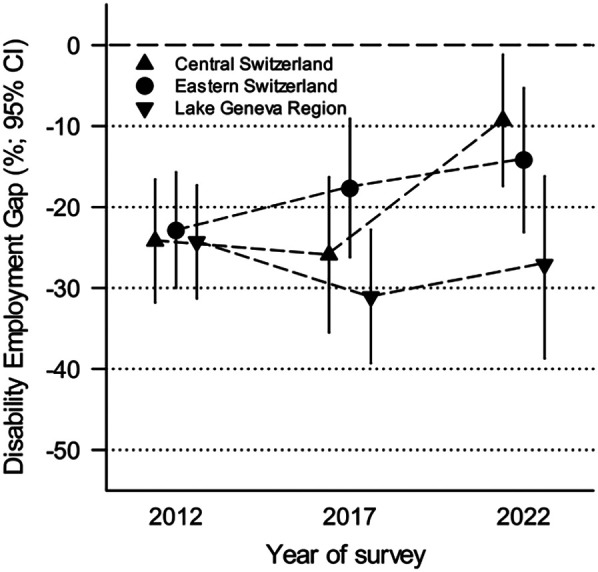
Trend in disability employment gap (% difference) over time for three indicative regions.

The DEG decreased over time for both males (−28% in 2012 to −16% in 2022) and females (−25% in 2012 to −18% in 2022), along with a decreasing LMP rate gap between males and females with SCI (Table 3). Similarly, the DEG decreased for all SCI severity levels (test of interaction: *x*^2^ = 42.3, df = 11, *p* < 0.001), with a greater decrease among those with more severe SCI (complete tetraplegia: 2012: −37%, 2022: −25%) (Table 3, Figure 1). The DEG changed over time for all groups of education (test of interaction: *χ*² = 83.11, df = 5, *p* < 0.001); it decreased for individuals with the most years of education (≥17 years: 2012: −13%, 2022: −9%), whereas it increased slightly for those with the fewest years of education (0–9 years: 2012: −48%, 2022: −49%) (Table 3, Figure 1). A decline in DEG over time was observed across most regions of Switzerland, except the Lake Geneva region where the DEG increased (2012: −24%, 2022: −27%). Notable improvements in DEG were observed in Eastern Switzerland (2012: −23%, 2022: −14%) and Central Switzerland (2012: −24%, 2022: −9%) (Table 3, Figure 2).

**Table 3:** Marginal DEGs over time for the SCI population, adjusted for sociodemographic and SCI characteristics.

Parameters	2012	2017	2022
	Gap (%), 95% CI	Gap (%), 95% CI	Gap (%), 95% CI
Overall	−25.9 (−28.8 to −23.1)	−22.3 (−25.6 to −19.0)	−17.3 (−20.8 to −13.7)
Sex			
Male	−27.7 (−33.4 to −21.9)	−24.9 (−31.8 to −18.0.)	−16.2 (−22.3 to −10.1)
Female	−25.4 (−28.7 to −22.1)	−21.7 (−25.5 to −17.9)	−17.8 (−22.1 to −13.)
Age (years)			
16–24	−25.6 (−40.4 to −10.7)	−24.9 (−48.6 to −1.2)	−43.3 (−69.2 to −17.4)
25–39	−22.6 (−28.5 to −16.7)	−17.5 (−24.9 to −10.0)	−15.8 (−22.9 to −8.7)
40–54	−27.7 (−32.0 to −23.4)	−23.9 (−28.9 to −18.9)	−15.4 (−21.1 to −9.7)
55–63/64	−27.3 (−32.6 to −22.0)	−23.7 (−29.4 to −18.0)	−21.4 (−27.3 to −15.5)
SCI severity			
Incomplete paraplegia	−22.3 (−27.1 to −17.5)	−11.8 (−17.0 to −6.6)	−15.5 (−20.9 to −10.1)
Complete paraplegia	−23.5 (−28.2 to −18.7)	−19.9 (−25.9 to −14.0)	−15.7 (−22.1 to −9.2)
Incomplete tetraplegia	−31.1 (−37.9 to −24.3)	−33.8 (−41.4 to −26.2)	−19.1 (−26.9 to −11.2)
Complete tetraplegia	−37.0 (−45.7 to −28.2)	−37.2 (−46.9 to −27.5)	−25.0 (−36.9 to −13.1)
Education (years)			
0–9	−48.1 (−59.1 to −37.1)	−53.6 (−67.0 to −40.2)	−49.2 (−71.4 to −27.0)
10–12	−39.5 (−45.6 to −33.3)	−34.9 (−43.0 to −26.9)	−34.2 (−43.8 to −24.6)
13–16	−21.3 (−25.4 to −17.2)	−21.5 (−26.5 to −16.6)	−15.2 (−20.1 to −10.3)
≥17	−13.2 (−19.3 to −7.2)	−7.9 (−13.6 to −2.2)	−9.0 (−15.1 to −2.8)
Region of residence			
Midland region	−27.4 (−32.9 to −21.9)	−17.8 (−23.7 to −12.0)	−17.5 (−24.5 to −10.5)
Northwestern Switzerland	−29.5 (−37.1 to −21.8)	−25.1 (−34.6 to −15.5)	−19.2 (−28.4 to −10.0)
Zurich	−27.4 (−36.7 to −18.1)	−21.7 (−32.0 to −11.3)	−14.7 (−23.7 to −5.8)
Eastern Switzerland	−22.9 (−30.0 to −15.7)	−17.7 (−26.2 to −9.1)	−14.2 (−23.1 to −5.3)
Central Switzerland	−24.2 (−31.8 to −16.6)	−25.9 (−35.5 to −16.3)	−9.3 (−17.4 to −1.2)
Ticino	−22.4 (−36.7 to −8.1)	−19.6 (−36.5 to −2.7)	−12.0 (−25.1 to 1.1)
Lake Geneva Region	−24.3 (−31.3 to −17.3)	−31.1 (−39.3 to −22.8)	−27.1 (−38.0 to −16.2)

CI, confidence interval; DEG, disability employment gap (in %); SCI, spinal cord injury.

## Discussion

The present study provides contemporary evidence regarding the LMP development in persons with SCI compared to the Swiss general population. This evidence indicates improving LMP opportunities for persons with SCI in Switzerland over the past decade, while also revealing that the LMP rate remains considerably lower than that of the general population. The DEG for the SCI population in Switzerland (17%) is lower compared to other high-income countries in Europe, such as Germany (33%), Norway (23%), and the Netherlands (24%) (5). Although data on national DEGs are available, DEGs segregated by socio-demographic characteristics and region within countries are lacking. Over the past decade we have found a decrease in DEGs, especially in individuals with tetraplegia, those with higher levels of education, and those residing in Central, Eastern and Southern Switzerland. In contrast, we found slightly increasing DEGs in the Southwestern Switzerland and in individuals with lower levels of education, suggesting that LMP opportunities for those affected are unequally distributed within the SCI population and across different regions in Switzerland.

A decline in the DEG may be attributable to improved public awareness and attitudes towards LMP among people with disabilities. However, in Switzerland, the overall DEG improvement in persons with impairments was only 0.9%, which is considerably lower than that in the SCI population (25). Thus, the decrease in DEG seems to be SCI-specific, potentially related to recent advancements of specialized vocational integration programs for persons with SCI, which are funded by most cantonal disability insurance offices in Switzerland.

The DEG decline in the SCI population may also be related to a rising demand for employees in less physically but more cognitively demanding jobs (26), as well as recent advances in the use of assistive technologies or devices, which can enhance the work-related functioning of individuals with severe physical impairments (27). The use of advanced technologies can support people in performing physical tasks with minimal physical effort, thus increasing the accessibility of jobs and workplaces (28), and enabling more individuals with physical impairments to return to and stay in the labor market. Finally, advancements in medical treatment and improved rehabilitation services for SCI may have contributed to the improved LMP in those affected (29).

A higher level of education increases the prospect of LMP for people with SCI, thereby contributing to a decreased DEG. Several studies in the SCI context have shown that individuals with higher educational levels are more likely to be in paid employment (7, 12, 30). A non-peer-reviewed study in the UK showed that investing in the education of persons with disabilities would reduce the DEG by 29%, and found the greatest decline in DEG for the least qualified (31). Similarly, a SwiSCI-based study found that promoting education could increase the LMP rate in people with SCI by up to 5% (9). In line with existing evidence that vocational education programs can help strengthen the work-related competencies and skills of persons with disabilities, thereby increasing their employability and promoting employment stability, our findings indicate increasing investment and coverage in education programs by disability insurance for those affected, to improve LMP prospects in the SCI population. The current social security regulations for providing vocational retraining in Switzerland are rather restrictive, as individuals without a Swiss education diploma are not entitled to receive a vocational retraining upon the onset of a disability (32). Individuals with a low level of education are thus at increased risk of compounded disadvantage in today's labor market due to the increase in cognitively demanding jobs (26), especially if they do not have a Swiss nationality. A recent SwiSCI-based study found that foreign nationals with SCI have a lower likelihood of LMP than Swiss nationals (9). Although this study is focused on SCI, these findings suggest a need for social policy reforms to ensure equitable access to vocational retraining, regardless of nationality or educational background, to support those most at risk of exclusion from the labor market.

Our study found regional disparities in the DEG development, and similar findings were reported in a recent British study (33). In Switzerland, these regional DEG disparities may reflect unequal access to SCI-specialized vocational integration measures in different regions of the country, including structural differences in the delivery of vocational integration services, insufficient funding of some cantonal disability insurances, and different levels of sensitivity to SCI-specific needs and difficulties among disability insurance advisors. The Swiss Paraplegic Center, located in Central Switzerland provides specialized inpatient and outpatient vocational integration services for individuals with SCI. Initiating vocational integration services early during inpatient rehabilitation can facilitate the retention of pre-injury employment or establish a foundation for subsequent vocational integration services, including job placement and vocational retraining if a career change is necessary (34). Research indicates that early initiation of vocational integration increases the likelihood of returning to work post-injury (35, 36). Conversely, individuals from Northwestern and Southwestern (Lake Geneva region) Switzerland typically complete their initial rehabilitation in facilities in their respective regions, where vocational integration services are not SCI-specific. Cantonal disability insurance offices in these regions are likely to refer individuals with SCI to non-SCI specialized local vocational integration providers, often with a delay following initial rehabilitation. Ensuring access to comprehensive SCI-specialized vocational integration services throughout the continuum of care (inpatient, outpatient, on-the-job) for all individuals with SCI across Switzerland can help address regional inequalities in LMP opportunities. At the national level, awareness campaigns targeting employers and insurance companies could help dispel misconceptions and encourage employment of more individuals with SCI. The implementation of employment quota systems or tax abatement policies may incentivize employers to hire persons with disabilities and potentially enhance the LMP of individuals with SCI. However, it is crucial to address the unintended consequences of such policies (e.g., tokenism or stigmatization) to promote a genuinely inclusive labor market (37).

Evidence of the longitudinal development of DEGs between persons with SCI and a country's general population is currently lacking, which limits the comparison of our findings with those of other studies. Since the DEG is commonly used as an indicator of social progress in the 27 European Union member states (4), benchmarking the LMP of individuals with disabilities to the general population is crucial for measuring social inequality and monitoring the progress of social policies aimed at supporting the LMP of persons with disabilities. While the current scarcity of evidence on DEG in the SCI context impedes the formulation of evidence-based LMP policies and the monitoring of their progress, our study advocates the use of longitudinal surveys as a strategy to enhance and improve the periodic collection of LMP data among persons with disabilities.

### Strengths and limitations

One of the strengths of our study is that it is the first to assess the longitudinal development of the DEG within the context of SCI, while accounting for SCI-related and socio-demographic characteristics, as well as within-person temporal changes. Therefore, this study provides reliable inference in support of regional evaluation and potential modification of vocational integration policies within the context of SCI. However, our study has some limitations. Primarily, the research may be affected by sampling bias due to self-selection or longitudinal attrition, potentially resulting in an overrepresentation of healthier or economically more active individuals, or dropout of individuals who differ systematically from those retained in the study. Additionally, self-reporting of LMP and their predictor variables may be subject to reporting bias, including information bias or social desirability bias. For example, participants may misrepresent their LMP status by claiming employment due to the negative societal perceptions associated with unemployment. Such response bias can lead to distorted estimates and inferences. Moreover, the findings relating to education should be interpreted with caution, as in the context of SCI, an increased duration of education may not necessarily indicate a higher level of educational attainment, given that the level of supplementary vocational re-education may be equivalent to the pre-SCI education level.

### Policy and future research implication

A longitudinal assessment of DEGs in comparison to the general population is essential for monitoring employment disparity and identifying policy intervention targets to improve labor market prospects for individuals with disabilities. The implementation of periodic national and regional LMP assessments can facilitate further evaluation and continuous improvement of LMP opportunities, thereby decreasing the DEG. Our findings revealed disparities in DEGs associated with injury severity, education level and geographic region. These results underscore the necessity of ensuring a comprehensive package of specialized vocational integration services for people with SCI across all geographic regions, with particular emphasis on vulnerable groups. Specialized vocational services can optimize individuals' employability and long-term resilience, thus enhancing their prospects for success in the labor market. Moreover, efforts and resources should be allocated to expanding the availability of quality employment opportunities. In this context, quality employment refers to employment situations that are commensurate with employees' training and experience, compatible with their health requirements, and appropriately remunerated. Such employment situations are likely to contribute to the sustainability of LMP. Regional disparities in DEGs in Switzerland can be partly explained by regional differences in availability and accessibility of vocational integration services, which affect the speed of the processes at the respective cantonal disability insurance offices. Additionally, cantonal disability insurance offices differ in their procedures for granting vocational integration services to individuals with disabilities. Beyond this, there is currently no evidence to suggest that variations in local disability policies could account for regional differences in LMP rates. More in-depth research is required to identify the factors contributing to regional disparities and to ensure equitable LMP opportunities across all regions of Switzerland.

## Conclusions

Our study demonstrated that LMP rates among individuals with SCI have improved relative to the general Swiss population over the past decade, particularly for individuals with tetraplegia, those with higher levels of education, and those residing in the Eastern and Central region of the country. Nevertheless, the observed increase in the DEG among people with lower educational attainment and those living in the Southwestern region of the country highlight a particular urgency for targeted vocational integration efforts at both the demographic and regional levels. These efforts may include the promotion of vocational education for severely injured individuals with lower educational qualifications and increasing the equality of access to SCI-specialized vocational integration services across all geographical regions of the country.

## Data Availability

The datasets presented in this article are not readily available because the Swiss New Federal Act on Data Protection and the European General Data Protection Regulation prohibit the public availability of individual data due to privacy considerations. Request to access the data should be directed to the corresponding author at mahesh.sarki@paraplegie.ch.

## Footnotes

^1^Sheltered work means individuals with impairments working at protected workplaces and those being unable to compete in the general labor market.
